# Pregnant women's awareness of sensitivity to cold (*hiesho*) and body temperature observational study: A comparison of Japanese and Brazilian women

**DOI:** 10.1186/1756-0500-4-278

**Published:** 2011-08-05

**Authors:** Sachiyo Nakamura, Sueli MT Ichisato, Shigeko Horiuchi, Taeko Mori, Masako Momoi

**Affiliations:** 1 Keio University,4411 Endo, Fujisawa, Kanagawa 252-8330, Japan; 2 Nursing Department, State University of Maringa, Av. Colombo, 5790 Bloco 1, Sala 15, Maringa, Parana, Brazil; 3 St. Luke's College of Nursing and St.Luke's Birth Clinic, 10-1, Akashi-cho, Chuo-ku, Tokyo 104-0044, Japan; 4 Mori Birth Center, 4-13-3, Mikageishi-machi, Higashinada-ku, Kobe 658-0045, Japan; 5 St. Mary's College, 422, Tsubukuhonmachi, Kurume 830-8558, Japan

## Abstract

**Background:**

Sensitivity to cold (*hiesho*) is a serious health problem in Japan, yet it is minimally understood within Western cultures. The purpose of this study was to clarify the divergence between pregnant Japanese woman living in Japan and pregnant Brazilian women living in Brazil in awareness of *hiesho *and differences between core body and peripheral temperatures.

**Methods:**

The subjects of this study were 230 pregnant Japanese women living in Japan and 200 pregnant Brazilian women living in Brazil. Data was collected in June/July and November 2005 in Japan and from October 2007 to February 2008 in Brazil. The survey methods consisted of measurement of deep body temperatures and questionnaires.

**Results:**

67.0% of Japanese women and 57.0% of Brazilian women were aware of *hiesho*, which showed a significant difference between the Japanese and Brazilian women (p = 0.034). The difference between forehead and sole temperatures was 2.0°C among Japanese and 2.8°C among Brazilians in June-July (p = 0.01). But in November the difference between those temperatures was 5.2°C among Japanese and 2.8°C among Brazilians (p < 0.001).

**Conclusions:**

There are differences between Japanese and Brazilians both in awareness of *hiesho *and in body temperatures.

## Background

*Hiesho*, the Japanese word for "sensitivity to cold," is a serious health problem in Japan, adversely affecting over 50% of Japanese women [1-4]. Oriental medicine holds that "*hiesho *lies at the root of all ailments," causing all types of illnesses, and that it is a condition that should be treated [5]. In the research of Miyamoto et al., Japanese women experienced cold hands and feet even during the summer, and this phenomenon was reported to be significantly higher among those who were aware of *hiesho*[6]. Despite the fact that *hiesho *is a well-known concept in Japan and throughout Asia, even major medical textbooks in the West make no mention of *hiesho *[7,8].

Nakamura [9] used Rodger's concept analysis to clarify the definition of *hiesho*, and three attributes of the concept "*hiesho*" were identified. Accordingly, *hiesho *was defined as a state where there exists a difference between core body temperature and peripheral temperature, and slow recovery of the peripheral temperature in a warm environment, with the subject usually having an awareness of *hiesho*. However, most of the articles dealing with *hiesho *she found for the analysis were written by Japanese researchers, and it was suggested that the concept "*hiesho*" did not exist in the West. As a result of Nakamura's study on Japanese pregnant women [4], it was found that *hiesho *was related to minor health problems and the daily activities of Japanese women.

On the other hand, the study by Nakamura et al. [10] on Brazilian pregnant women did not recognize any relationship between *hiesho *and daily activities, even though more than 50% of Brazilian women were aware of *hiesho*. In other words, they did not feel *hiesho *as discomfort and it was suggested that even increased awareness of *hiesho *did not worsen minor health problems related to *hiesho*.

From the above, it is understood that there is a big difference between Japanese and Brazilians as far as *hiesho *is concerned.

This study was conducted on Japanese pregnant women living in Japan and Brazilian pregnant women living in Brazil for elucidating the divergence between women in these two countries in awareness of *hiesho *and differences between core body and peripheral temperatures.

The author believes this research will be helpful in explaining and establishing the concept of *hiesho*, and thus contribute to an important area of cultural anthropological study of Japanese people.

The operational definition of terms used in this study is as follows. Core body temperature: Temperature of the internal deep tissues less affected by ambient environmental temperature [11]. *Hiesho *(sensitivity to cold): A medical condition characterized by differences between core body temperature and peripheral temperature where the peripheral temperature is slow to increase, even in warm ambient temperatures. In many cases, it is accompanied by an awareness of *hiesho *[9]. Awareness of *hiesho*: Determined by the answer to the question "Are you sensitive to cold?" in the Scale for Determining *Hiesho *(Terasawa) [3].

## Methods

### Subjects of study

The study was carried out among 230 Japanese women (Nakamura 2008) and 200 Brazilian women (Nakamura et al. 2010) who agreed to participate and who met the following criteria at the time of recruitment: 1) in the 20th or later week of pregnancy; 2) no complications affecting the pregnancy or body temperature (endocrine disorders, autonomic nervous system disorders, hypertension, heart disease, liver disease, kidney disease, psychiatric illness, etc.); and 3) normal progress of pregnancy.

Concerning 1), there is a physiological rise in body temperature until the fourth month of pregnancy, believed to be caused by the effects of progesterone coming from the corpus luteum, which increases metabolism. After the fourth month, body temperature gradually drops, returning to a normal level around the sixth to seventh month and remaining at that level until delivery [12]. Therefore, this research targeted subjects past the 20th week of pregnancy, when body temperature has stabilized.

This study is a reanalysis of part of previously published data. They were reanalyzed to meet the purpose of this study [4,10].

### Study Setting

Japan has four distinct seasons, whereas seasonal variations in Brazil less pronounced. This study compared Japanese and Brazilian pregnant women by dividing the research period in Japan between summer and late autumn.

In Japan, data was collected in June-July and November 2005, at a maternity clinic in Tokyo. The study period in Brazil was from October 2007 to February 2008, with data collected from one university hospital and four regional health centers in Maringa, Parana State.

### Data Collection

To ensure reliability and appropriateness, care was taken to use consistent data collection methods throughout. To measure deep body temperatures, probes were attached to the middle of the subjects' forehead and the middle of the sole of the foot. Miura, using thermography to measure surface skin temperature, reported that acclimatization time took 10 minutes [13]. In this study, taking the ambient temperature into consideration, the women's temperatures were measured after they had spent at least 20 minutes in a measuring room where a constant temperature was maintained. After their temperatures had been measured, the women were given questionnaires to complete and hand back to the researcher.

### Instrument (Deep Body Temperature)

A Japanese-made Core-Temp® CTM-205, Terumo Corp., Tokyo, Japan, a reliable and minimally invasive thermometer using zero-heat-flow technology, was used for measuring deep body temperatures from surface skin. Deep body temperatures were measured on the forehead and the sole of the foot, to obtain temperature differences between these two parts of the body. Tsuji reported that forehead and abdomen temperatures correspond to deep body temperature called core body temperature, whereas temperatures of the palm of the hand and the sole of the foot are peripheral temperatures and correspond to the shell temperature. Forehead temperature is believed to reflect brain temperature, and Tsuji noted that it was close to pulmonary artery blood temperature [14,15]. This indicates that among deep body temperatures, forehead temperature is close to the core body temperature, and that the lowest temperature is the sole temperature. Therefore, the forehead temperature measured in this study reliably reflects core body temperature. Yamazaki [16] reported that there were no temperature differences between the left and right sides of the body; subjects' temperatures were measured on either side of the body where a probe could be effectively attached.

### Awareness of *hiesho*

Subjects were asked to fill in Terasawa [3]'s questionnaire to determine their awareness of *hiesho*. According to a preceding study, "awareness of *hiesho*" and the answer to the question "Are you sensitive to cold?" in the Scale for Determining *Hiesho *(Terasawa) coincided for 80.0% of Japanese and 79.9% of Brazilians, showing significant correlation. In other words, it was possible to determine awareness of *hiesho *based on one item in the scale -- whether the individual believed that she was or was not sensitive to cold.

Concerning the survey in Brazil, in order to avoid misunderstandings caused by language differences, a specialist in Portuguese and Japanese was asked to translate the questionnaire and explanatory documentation into Portuguese. In addition, a different specialist carried out the back-translation. Thus, accuracy was verified.

### Ethical considerations

The study in Japan was conducted with the approval of the Ethics Review Committee of St. Luke's College of Nursing (05-012). In Brazil, the research representative entered into an international cooperation agreement with the university to which research collaborators belong (Parana State official gazette no. 7664). The Ethics Review Committee of St. Luke's College of Nursing (07-029) and Maringa University, Parana State (PARECER n.298/2007), and the Ethics Examination Committee of the National Ethics Council (PARECER n. 54/2008) granted approval.

Individuals willing to cooperate in this research project were given both written and oral explanations of the research, and when they agreed to participate, were asked to sign an informed consent form. If they decided that they wanted to drop out of the study after giving their consent, they were asked to sign a form saying they would no longer participate. Due care was paid to ethical considerations, in order to protect the human rights of the subjects.

### Data Analyses

Differences in temperatures at the forehead and on the sole of the foot were compared, using a t test. In all cases, standard deviation, 95% confidence interval (CI value), degrees of freedom and p values were calculated, with a 5% significance level. Statistics software SPSS Statistics 19.0 was used for statistical analysis.

BMI values (kg/m^2^) were classified according to the WHO definition as follows: underweight <18.50, normal range 18.50-24.99, overweight **≥**25.00, and obese **≥**30.00 [17].

## Results

The subjects of this study were 235 pregnant Japanese women living in Japan and 206 pregnant Brazilian women living in Brazil. Out of the subjects, five Japanese and six Brazilian women withdrew prior to the study. Ultimately, 230 Japanese and 200 Brazilians became the subjects of the analysis.

Among the Japanese subjects, 130 women were surveyed in June-July and 100 in November, for a total of 230. The total number of Brazilian women surveyed was 200.

Characteristics of all subjects were compared as shown in Table 1.

**Table 1:** Demographic Characteristics of Participants

	Japanese (n = 230)	Brazilian (n = 200)			
	**Mean (SD)/n (%)**	**Mean (SD)/n (%)**	**t value/χ2 value**	**df**	**p value**
Age	31.8(4.3)	26.2(5.7)	-11.15	373.79	<0.001*
Gestational age(weeks)	28.5(5.9)	29.3(5.8)	1.40	428	0.16
Elapsed time after meal intake(hour)	3.0(2.6)	3.1(3.1)	4.42	422.53	<0.001*
Body height(cm)	159.1(5.2)	161.3(6.3)	3.93	386.06	<0.001*
Body weight(kg)	57.3( 7.8)	70.97(13.1)	12.92	315.00	<0.001*
BMI Mean (SD) body mass index (kg/m2)					
Underweight	35.0(15.2)	8.0(4.0)	119.6	3	<0.001*
Normal range	140.0(60.9)	46.0(23.0)			
Overweight	34.0(14.8)	41.0(20.5)			
Obese	21.0(9.1)	105.0(52.5)			
Parity					
Primipara	132.0(57.4)	73.0(36.5)	18.72	1	<0.001*
Pluripara	98.0(42.6)	127.0(63.5)			
Occupation					
Homemaker	169.0(73.5)	72.0(36.0)	109.28	2	<0.001*
Company worker	38.0(16.5)	128.0(64.0)			
Other	23.0(10.0)	0.0(0)			

1) Age,Gestational age(weeks),Elapsed time after meal intake(hour),Body height(cm),Body weight(kg):t-test/Mean (SD), t-value

2) MI,Parity,Occupation:chi-square test,n (%),χ2-value

The average age of the Japanese was 31.8 years (SD4.3), older than the Brazilians' average of 26.2 years (SD5.7) (p < 0.001). Regarding obesity of Japanese based on average BMI (kg/m^2^), "normal range" accounted for the largest portion: 60.9% (140 persons), followed by "underweight: 15.2% (35 persons), while among Brazilians, "obese" accounted for 52.5% (105 persons), followed by "normal range" for 23.0% (46 persons). In other words, Japanese women were thinner than Brazilian women (p < 0.001).

### Comparison of subjects' backgrounds in *hiesho *in pregnant women (Table 2)

**Table 2:** Demographic Characteristics of Participants by Awareness of Sensitivity to Cold (*Hiesho*)

	Awareness of *hiesho *(N = 268)				
	**Japanese (n = 154)**	**Brazilian (n = 114)**			

	**Mean (SD)/n (%)**	**Mean (SD)/n (%)**	**t value/χ2 value**	**df**	**p value**
Age	31.8(4.3)	25.8(5.5)	9.76	206.56	<0.001*
Body height(cm)	158.7(5.4)	161.0(6.2)	-3.32	266	0.001*
Body weight(kg)	55.9( 6.9)	68.9(12.4)	-10.11	266	<0.001*
BMI Mean (SD) body mass index (kg/m2)					
Underweight	27.0(17.5)	6.0(5.3)			
Normal range	101.0(65.6)	35.0(30.7)	72.31	3	0.001*
Overweight	16.0(10.4)	18.0(15.8)			
Obese	10.0(6.5)	55.0(48.2)			
Parity					
Primipara	90.0(54.8)	44.0(38.6)	10.32	1	0.001*
Pluripara	64.0(45.2)	70.0(61.4)			
Occupation					
Homemaker	112.0(72.7)	36.0(31.6)	76.77	2	0.001*
Company worker	26.0(16.9)	78.0(68.4)			
Other	7.0(9.4)	0.0(0)			

1)Age,Body height(cm),Body weight(kg):t-test/Mean (SD), t-value

2)BMI,Parity,Occupation:chi-square test,n (%),χ2-value

Characteristics of pregnant women with awareness of *hiesho *were compared.

The average age of the Japanese was 31.8 years (SD4.3), older than the Brazilians' average of 25.8 years (SD5.5) (p < 0.001). Regarding obesity of Japanese based on average BMI (kg/m^2^), "normal range" accounted for the largest portion: 65.6% (101 persons), followed by "underweight" 17.5% (27 persons), while among Brazilians, "obese" accounted for 48.2% (55 persons), followed by "normal range" for 30.7% (35 persons). In other words, Japanese women with *hiesho *were thinner than Brazilian women with *hiesho *(p = 0.001). Ninety Japanese were primiparae (54.8%), while among Brazilians with *hiesho *70 were multiparae (61.4%) (p = 0.001).

### Comparison of Japanese and Brazilian pregnant women in awareness of *hiesho *(Table 3)

**Table 3:** Awareness of *hiesho *: comparison between Japanese and Brazilians

	Awareness of *hiesho*	
	Aware of *hiesho(*n/%)	Not aware of *hiesho *(n/%)
Japanese (n = 230)	154(67.0)	76(33.0)

Brazilian (n = 200)	114(57.0)	86(43.0)

chi-square value = 4.52 df = 1p = 0.034

67.0% of Japanese women (154 persons) and 57.0% of Brazilian women (114 persons) were aware of *hiesho*. In other words, 33.0% of Japanese women (76 persons) and 43.0% of Brazilian women (86 persons) were not aware of *hiesho *and the concordance rate was 80.9%. Results of a chi-square test also showed a significant difference between the Japanese and the Brazilian women (p = 0.034).

In short, Japanese women were significantly more aware of *hiesho *than Brazilian women.

### Comparison of differences between forehead and sole temperatures among women with awareness of *hiesho*

The deep body temperatures of Japanese and Brazilian pregnant women with awareness of *hiesho *were compared. The Japanese subjects (n = 88) were measured in June-July and the Brazilians (n = 114) in October-February (Table 4). Average outdoor temperature at the time of the study in Japan was 25.0°C and in Brazil 23.5°C.

**Table 4:** Comparison of Japanese and Brazilian deep body temperature characteristics by outdoor seasonal temperature (June/July)

	Japanese (n = 88) June/July = 25°C	Brazilian (n = 114) October - February = 23.5°C	t value	df	p value	mean difference	95% CI
	Mean (SD)	Mean (SD)					
Forehead temperature (°C)	36.4(0.30)	36.3(0.33)	-0.25	200	0.80	-0.01	-0.10-0.08
Sole temperature (°C)	34.4(1.67)	33.5(2.68)	-2.88	191.98	0.004	-0.89	-1.50--0.28
Difference (°C)	2.0(1.69)	2.8(2.68)	2.85	192.80	0.01	0.88	0.27-1.49

For Japanese in June-July, the mean forehead temperature of women with awareness of *hiesho *was 36.4°C, compared to 36.3°C for Brazilian women, which was not a significant difference (p = 0.8). The mean sole temperature of Japanese women was 34.4°C, compared to 33.5°C for Brazilian women, showing a significant difference (p = 0.004). The mean difference between forehead and sole temperatures among Brazilian women was 2.8°C, significantly larger than the 2.0°C for Japanese women (95% CI, 0.27 to 1.49; p = 0.01).

Additionally, data collected on Brazilians (n = 114) in October-February was compared with that of Japanese (n = 66) monitored in November (Table 5). As a result, the mean forehead temperature of Japanese women with awareness of *hiesho *was 36.4°C, compared to 36.3°C for Brazilian women, which was not a significant difference (p = 0.28). The mean sole temperature of Japanese women was 31.2°C, compared to 33.5°C for Brazilian women, showing a significant difference (p < 0.001). The mean difference between forehead and sole temperatures among Japanese was 5.2°C, significantly larger than the 2.8°C among Brazilians (95% CI, -3.19 to -1.61; p < 0.001). Average outdoor temperature at the time of the study in Brazil was 23.5°C and in Japan 13.3°C.

**Table 5:** Comparison of Japanese and Brazilian deep body temperature characteristics by outdoor seasonal temperature (November)

	Japanese (n = 66) November = 13.3°C	Brazilian (n = 114) October-February = 23.5°C	t value	df	p value	mean difference	95% CI
	Mean (SD)	Mean (SD)					
Forehead temperature (°C)	36.4(0.37)	36.3(0.33)	-1.08	178	0.28	-0.06	-0.16-0.05
Sole temperature (°C)	31.2(2.47)	33.5(2.68)	5.81	178	<0.001	2.34	1.55-3.14
Difference (°C)	5.2(2.40)	2.8(2.68)	-6.01	178	<0.001	-2.4	-3.19--1.61

## Discussion

### Differences in awareness of *hiesho*

It became clear that the Japanese had more awareness of *hiesho *than the Brazilians. Based on the preceding study, one reason for this was that *hiesho *caused discomfort to Japanese women, whereas Brazilian women did not feel as uncomfortable even though they were aware of *hiesho *[4,10]. Further, in terms of daily life, Brazilian women are not in the habit of using air-conditioning, whereas Japanese often spend time in air-conditioned places. In research by Miyamoto et al, it was reported that Japanese women felt cold in their extremities even in the summer, and that this was significantly higher (p < 0.01) among those who reported awareness of *hiesho*, due to the effects of their living environment, such as use of air-conditioning [6]. These differences in lifestyles probably amplified the Japanese pregnant women's awareness of *hiesho. *Further, culturally, Japanese are said to be methodical, delicate, and highly sensitive [18]. By comparison, according to Kubota, Brazilians have more relaxed personalities and weaker awareness toward childbirth preparation [19]. These differences in national temperament or ethnicity may be one reason for differences in awareness of *hiesho*.

### Relationship between awareness of *hiesho *and differences in deep body temperatures

By comparing differences between forehead and sole temperatures of subjects with awareness of *hiesho*, it was found that differences in deep body temperatures were reflected in awareness of *hiesho *among both the Brazilians and the Japanese. Therefore, this indicated that *hiesho *is not a condition particular to Asians; it also exists among Westerners. A comparison between Brazilians and the Japanese monitored in June and July revealed that the Brazilians had a significantly larger difference in deep body temperature. Additionally, comparing Brazilians to the Japanese monitored in November, the Japanese showed a significantly larger difference in deep body temperature compared to the Brazilians. In Brazil, the average outdoor temperature was 23.5°C when the women were monitored. In Japan, outdoor temperatures were 25.0°C in June and July, and 13.3°C in November. Therefore, in the comparison between Brazilians and the Japanese monitored in November, as one would expect, the reason for the significantly larger difference among the Japanese was probably the outdoor temperature lower by 10°C.

With regard to the relationship between body temperature and outdoor temperature, Imai et al reported that on a seasonal basis, an overwhelmingly large number of Japanese women were aware of *hiesho *in winter (78.9%) [20]. Arawaka and Miura found that seasonal temperature changes were experienced most strongly in the periphery and reported that peripheral temperature was lower in winter than in summer [13,21]. These findings also indicated that temperatures in Brazil, where the climate is sub-tropical and transitions between the seasons are less clear than in Japan, remained relatively stable year-round compared to Japan, but that differences in deep body temperatures became larger if the outdoor temperature declined. In other words, the comparison between the Brazilians and the Japanese with awareness of *hiesho *indicated that differences between forehead and sole temperatures would vary depending on outdoor temperature (season).

## Conclusions

It became clear that Japanese women were more aware of *hiesho *than Brazilian women.

By comparing differences between core body and peripheral temperatures of pregnant women with awareness of *hiesho*, it was found that differences in deep body temperatures were reflected in awareness of *hiesho *among the Brazilians as well as the Japanese. Therefore, this indicated that *hiesho *is not a condition particular to Asians; it also exists among Westerners.

In addition, it was presumed that differences in deep body temperatures were affected by outdoor seasonal temperatures by comparing the differences in deep body temperatures between the Japanese monitored in June and July and the Brazilians, and between the Japanese monitored in November and the Brazilians

## Competing interests

The author declares that they have no competing interests.

## Authors' contributions

SN had full access to all data in the study, and took responsibility for the integrity of the data, the accuracy of the data analysis, and the decision to submit the paper for publication. SMTI and TM participated in the study design and acted as coordinators during fieldwork. SH was in charge of data analysis and acted as supervisor. MM helped draft the manuscript. All authors read and approved the final manuscript.
